# The HUMTICK study: protocol for a prospective cohort study on post-treatment Lyme disease syndrome and the disease and cost burden of Lyme borreliosis in Belgium

**DOI:** 10.1186/s13690-017-0202-z

**Published:** 2017-08-07

**Authors:** Laurence Geebelen, Tinne Lernout, Benoît Kabamba-Mukadi, Veroniek Saegeman, Hein Sprong, Steven Van Gucht, Philippe Beutels, Niko Speybroeck, Katrien Tersago

**Affiliations:** 1 Epidemiology of Infectious Diseases, Scientific Institute of Public Health (WIV-ISP), Rue Juliette Wytsmanstraat 14, 1050 Brussels, Belgium; 2 Laboratory of Medical Microbiology, Université Catholique de Louvain (UCL), Brussels, Belgium; 3 Department of Microbiology, University Hospitals Leuven, Leuven, Belgium; 4 Laboratory for Zoonoses and Environmental Microbiology, National Institute for Public Health and Environment (RIVM), Bilthoven, The Netherlands; 5 Viral Diseases, Scientific Institute of Public Health (WIV-ISP), Brussels, Belgium; 6 Centre for Health Economics Research & Modelling Infectious Diseases (CHERMID), Vaccine & Infectious Disease Institute, Faculty of Medicine & Health Sciences, University of Antwerp, Wilrijk, Belgium; 7 Institute of Health and Society (IRSS), Université catholique de Louvain, Brussels, Belgium

**Keywords:** Lyme borreliosis, Post-treatment Lyme disease syndrome, Disease burden, Disability-adjusted life year, Economic cost

## Abstract

**Background:**

In Belgium, different routine surveillance systems are in place to follow-up Lyme borreliosis trends. However, accurate data on the disease and monetary burden for the different clinical manifestations are lacking. Despite recommended antibiotic treatment, a proportion of Lyme patients report persisting aspecific symptoms for six months or more (e.g. fatigue, widespread musculoskeletal pain, cognitive difficulties), a syndrome now named “post-treatment Lyme disease syndrome” (PTLDS). Controversy exists on the cause, incidence and severity of PTLDS. This study aims to estimate the incidence of PTLDS in patients with Lyme borreliosis and to quantify the disease burden and economic costs associated with the different clinical manifestations of Lyme borreliosis in Belgium.

**Methods:**

The project is a prospective cohort study in which about 600 patients with an erythema migrans and 100 patients with disseminated Lyme borreliosis will be followed up. Questionnaires, including the SF-36 vitality and pain subscale, the Cognitive Failure Questionnaire and the EQ-5D-5L, will be used to collect information on acute and persisting symptoms and the impact on quality of life. Symptom frequency and severity will be compared with self-reported pre-Lyme health status, a control group and existing Belgian population norms. Additionally, information on the associated costs and possible risk factors for the development of PTLDS will be collected.

**Discussion:**

A study of the health burden will allow evaluation of the relative importance of Lyme borreliosis in Belgium and information on the economic cost will help to formulate cost-effective measures. There are only few prospective studies conducted estimating the incidence of PTLDS and even though discussion exists about the prevalence of subjective symptoms in the general population, a control group of non-Lyme borreliosis participants has often not been included.

**Electronic supplementary material:**

The online version of this article (doi:10.1186/s13690-017-0202-z) contains supplementary material, which is available to authorized users.

## Background

Lyme borreliosis is a multisystem infectious disease caused by bacteria from the *Borrelia burgdorferi* sensu lato complex. These spirochetes are transmitted by hard ticks, predominantly by *Ixodes ricinus* in Europe. Lyme borreliosis is the most prevalent arthropod-borne disease in Europe and North America [1, 2]. Asymptomatic infections are frequent [3–5]. If disease develops, it can cause a wide range of symptoms. The most common clinical manifestation is an erythema migrans (EM), a red expanding rash which occurs in 60 to 95% of symptomatic infections, within days to weeks after the tick bite [6–11]. In this early disease stage, flu-like symptoms (e.g. fever, headache, fatigue, arthralgia’s, myalgia’s) may accompany EM or appear separately. If an infection is not treated with appropriate antibiotics, it can possibly evolve to disseminated Lyme borreliosis (weeks, months or years after the tick bite) affecting the skin, the nervous system (Lyme neuroborreliosis), the joints (arthritis) or more rarely, the heart or the eyes [1, 12]. Despite adequate antibiotic treatment, it has been reported that some people still suffer from a range of non-specific symptoms after an infection: fatigue, widespread musculoskeletal pain or cognitive difficulties (e.g. memory problems, difficulties concentrating, problems finding words). If these symptoms persist for more than six months after treatment in correctly diagnosed Lyme patients and are of such severity that they influence the patient’s daily life, this is referred to as post-Lyme disease syndrome [2], later named post-treatment Lyme disease syndrome (PTLDS) [13–15].

The frequency of subjective symptoms after treatment for Lyme borreliosis reported in the literature varies widely; for early Lyme borreliosis (EM) it ranges between 5 and 20% [1, 13, 16–19] reaching 36% if included patients present systematic symptoms as well (e.g. flu-like symptoms) [14]. A review by Koedel et al. (2015) reported that PTLDS symptoms developed in 5–54% of Lyme patients that suffered from Lyme neuroborreliosis [16]. There is controversy and concern about the prevalence of these aspecific symptoms in the general population and although it is advised, only few studies included a non-Lyme borreliosis control group [17, 19–22]. The pathogenesis of the syndrome is still unclear, different possible explanations have been suggested (e.g. persistent infection, post-infective fatigue syndrome, natural response after treatment, autoimmune mechanisms, intercurrent conditions) but no conclusions can be made [15, 17, 23]. Several risk factors for the development of PTLDS have been hypothesized, including dissemination of disease, delayed diagnosis and treatment, more severe symptoms at diagnosis and the elevation of immune mediators (e.g. elevated CCL19) [15, 23–25].

It is thought that co-infections with other tick-borne pathogens such as *Anaplasma* spp. and *Babesia* spp. can exacerbate the clinical presentation of Lyme borreliosis: they can increase the severity of initial Lyme symptoms, causing unexplained leukopenia, thrombocytopenia or anemia, or high-grade fever after treatment [2, 26]. In the last years, other less known tick-borne pathogens have been found to circulate in Belgian ticks, amongst others, *Borrelia miyamotoi*, *Neoehrlichia mikurensis* and *Rickettsia* spp. [27–29]. It is unclear to what extent co-infections with these and other tick-borne pathogens may affect persisting symptoms after treatment of Lyme borreliosis [2, 30–35].

In Belgium, it is estimated that, every year, approximately 10.000 people consult a general practitioner (GP) with an erythema migrans and 200–300 people are hospitalized due to disseminated Lyme borreliosis [36, 37]. Although, up to now, no increasing trend in the Lyme borreliosis incidence has been observed (based on the long-term surveillance systems in place), the disease is assumed to represent a significant health and cost burden for the population and the health system in Belgium. This has however not yet been assessed. Lyme borreliosis and especially its possible long-lasting symptoms, even after treatment, remain a subject of controversy in Belgium, as in other parts of the world [2, 38]. In order to provide some answers to this debate, this prospective study is set up.

### Study objectives

The aim of the study is to evaluate the incidence of and possible risk factors for the development of post - treatment Lyme disease syndrome (PTLDS) and to estimate the disease burden and economic cost associated with the different clinical manifestations of Lyme borreliosis in Belgium.

## Methods

### Study design

The core of HUMTICK is a prospective cohort study with follow-up of patients with Lyme borreliosis and a non-Lyme borreliosis control group (in a 1:1 ratio). Questionnaires will be used to collect information on duration and severity of symptoms, health related quality of life, costs and possible risk factors for the development of PTLDS. At the end of the follow-up, a case-control study will be set up within a sub-cohort of EM patients, to analyze tick-borne co-infections as a possible risk factor for the development of PTLDS. In this case-control study, cases are defined as EM patients with PTLDS and controls (in a 1:2 ratio) as patients in which EM resolves without persisting symptoms. To search for the presence of these other tick-borne diseases a blood sample will be collected from all the patients in the EM sub-cohort at the moment of their diagnosis (acute infection). In addition to the prospective study, existing databases will be consulted to obtain supplementary data, necessary for the calculation of the full disease and cost burden of the different clinical manifestations of Lyme borreliosis.

### Study population

Two sub-cohorts will be enrolled in the cohort study; one cohort consisting of patients who consult a GP with an erythema migrans and a second consisting of patients with confirmed disseminated Lyme borreliosis (e.g. neuroborreliosis, Lyme arthritis, Lyme carditis) diagnosed by a specialist physician in a hospital. A non-Lyme borreliosis control group, matched for age and gender is added (in a 1:1 ratio) to allow comparison between the cohorts and the general population. Children (<18 years) and pregnant women will be excluded from the study. All participants will sign an informed consent form prior to inclusion. The case definitions for inclusion for cohort 1 and 2 are shown in Table 1.

**Table 1:** Case definitions for inclusion in the HUMTICK study (2016)

	Case definition
Cohort 1: EM	- Clinical presentation of EM, confirmed by a GP
Cohort 2: Confirmed disseminated Lyme borreliosis	- Positive polymerase chain reaction (PCR) or culture OR - Positive serology AND at least one clinical manifestation compatible with disseminated Lyme borreliosis (confirmed by the treating physician) [12] [see Additional file 1 for the specific description of this case definition]

### Participant enrollment

Participants are enrolled since June 2016 and up to August 2018 with follow-up ending no later than February 2019. For cohort 1 (EM), a network of approximately 200 GPs is currently being set up in areas in Belgium that are highly endemic for tick bites and Lyme borreliosis. All patients complying with the case definition are invited to participate, until inclusion of a sufficient sample. Patients with confirmed disseminated Lyme borreliosis (cohort 2) are enrolled through the hospitals linked to the Belgian National Reference Centers (NRC) for Lyme borreliosis: Cliniques Universitaires Saint-Luc-UCL and UZ Leuven (infectious diseases, neurology and rheumatology departments). If this is not sufficient to attain the desired sample size, other hospitals will be involved at a later stage (2017–2018). Participants of the non-Lyme borreliosis control group are selected by the patients in cohorts 1 and 2, among persons in their own environment with the same gender, comparable age (+/− 5 years) and no prior Lyme borreliosis diagnosis. The participants are included in the study after validation of the criteria.

### Sample size estimates and power calculation

For the sample size calculation of **cohort 1**, we assume that 12.5% of EM patients will develop PTLDS (mean of 5–20% [1, 13, 16–19]) in comparison with 5% [19] of the non-Lyme controls developing the same symptoms. For the sample size calculation of **cohort 2**, we assume 20% of disseminated Lyme patients developing PTLDS. With an alpha of 0.05, a power of 90 and a cohort-controls ratio of 1:1, this means that we need 285 EM patients in cohort 1 and 93 disseminated Lyme patients in cohort 2 (2 sided-test) to allow detection of a risk ratio﻿ (RR) of respectively 2.5 and 4.

However, to analyze the association between PTLDS and co-infections as a risk factor in the **case-control** study (set-up within cohort 1), we need 74 cases of PTLDS and 148 controls based on an alpha of 0.05, a power of 0.80, a case-controls ratio of 1:2 and the assumption of a less than medium effect size of 0.4 (2-sided test). This means that, if 12.5% of the EM patients develop PTLDS, we need to extend **cohort 1** to 592 EM patients in order to have 74 cases in the case-control study**.** Taking into account a reasonable loss to follow-up (± 25% over 2 years), 780 EM patients and 120 disseminated Lyme patients need to be included in the study.

### Data collection procedures

Data collection will start when the patient is diagnosed (T0) with Lyme borreliosis at the consultation with the GP (cohort 1) or in the hospital (cohort 2). During this consultation, the first part of the first paper questionnaire is filled in together with the treating physician (questions on comorbidity, acute symptoms, diagnosis and treatment). The second part of the questionnaire can be filled in by the patients themselves after the consultation (questions on socio-demographic parameters, general health before Lyme borreliosis, tick bite exposure, acute symptoms and costs). The patients’ follow-up questionnaires are completed online (or on paper if preferred by the patient) at different points in time: after 1 month, 3 months, 6 months, 12 months and 24 months. The period of patient follow-up depends on the moment of enrollment and will last maximum 24 months (patients enrolled in early study phase) and minimum 6 months (patients enrolled in final phase of enrollment). Control persons will be requested to fill in a questionnaire at inclusion, after six months and after one year. The blood sample for the patients of cohort 1 (EM) will be collected at T0.

#### Post-treatment Lyme disease syndrome (PTLDS)

PTLDS is defined (in line with the case definition for post-Lyme disease syndrome proposed by the Infectious Diseases Society of America (IDSA) [2, 13]) as the new onset of fatigue, widespread musculoskeletal pain and/or cognitive difficulties in patients diagnosed with and treated for Lyme borreliosis. Symptoms should occur within six months after the diagnosis, be present for more than six months (continuous or relapsing) and impact the patient’s daily life functioning (quality of life). Patients will be excluded if the symptoms are not new or when there is another explanatory cause for their symptoms.

To apply this case definition, the first patient questionnaire (T0) will assess the presence and severity of the subjective symptoms, as well as the impact on the patient’s life, before the onset of their Lyme borreliosis (during their “pre-Lyme” general health status). The post-treatment presence, severity and impact of the subjective symptoms will be assessed in the same way, in the different follow-up questionnaires (from 3 months onwards). This will allow comparison between the pre- and post-Lyme health status of the patient.

In order to assess the severity and impact more accurately, validated questionnaires will be used:

- The SF-36 is a self-report 36-item health survey (8 subscales) which has been used widely and was shown to have good reliability and validity [39–41]. In our study two subscales are used; the SF-36 vitality subscale (4 items) to assess fatigue and the SF-36 bodily pain subscale (2 items) to assess widespread musculoskeletal pain.
- The Cognitive Failure Questionnaire (CFQ), a 25-item questionnaire of which psychometric properties have been proven to be good, is used to evaluate cognitive difficulties [42, 43].
- The EQ-5D-5L, a standardized non-disease specific instrument to evaluate the health-related quality of life, developed by the EuroQol group association [44–46], is added to each questionnaire to assess the impact of the symptoms on the patient’s quality of life.
- The Global Activity Limitation Index (GALI), a single item instrument, is added to the questionnaires from 6 months onwards since it measures the presence of long-lasting (≥ 6 months) health related activity limitations [47–49].

The same questionnaires will be used for people from the non-Lyme borreliosis control group, to allow analysis of the incidence of the same symptoms in the general population. Furthermore, currently Belgian population norms exist for the SF-36 vitality, SF-36 bodily pain, the EQ-5D-5L and the GALI, CFQ norms are only available for the Dutch population [50–54].

Finally, the GPs of the patients in cohort 1 (EM) will fill in an online follow-up questionnaire, based on the patient’s medical file, after six and twelve months. Questions are asked about possible supplementary consultations, additional diagnoses, new treatments prescribed and laboratory results if requested. This will allow comparison between the patient’s and GP’s perspectives on the subjective symptoms.

#### Risk factors for PTLDS

To identify possible risk factors for the development of PTLDS, information on comorbidity, tick bite exposure, severity and duration of symptoms presented, prescribed treatment (type, duration) as well as socio-demographic variables (age, sex, education and employment status) will be collected during the complete study. The blood samples collected at T0 from EM patients will be tested, by means of a multiplex PCR, for the presence of *Anaplasma* spp.*, Rickettsia* spp.*, Neoehrlichia mikurensis, Borrelia miyamotoi* and *Babesia* spp. at the Laboratory for Zoonosis and Environmental Microbiology (RIVM), the Netherlands [55].

#### Disease burden

The Burden of disease will be expressed in Disability-Adjusted Life Years (DALYs), a summary health measure comprising both mortality and morbidity. DALYs measure the healthy life years lost due to illness and equal the sum of the years lived with disability (YLDs) and the years of life lost due to premature mortality (YLLs). The YLDs are calculated based on the occurrence, severity (disability weights (DW)) and duration of the disease health states [56–58]. An incidence approach will be used for the occurrence of the different clinical manifestations of Lyme borreliosis, where the incidences of EM and disseminated Lyme will be estimated based on existing literature and surveillance systems currently in place in Belgium (e.g. sentinel network of general practitioners, network of sentinel laboratories and minimal clinical data) [36, 37]. The cohort study will provide an estimate of the incidence of PTLDS, of the symptom durations for the different clinical manifestations as well as estimates of the group specific disability weights. The latter will be derived from the participants’ EQ-5D-5L responses: following the EQ-5D-5L user guide the EQ-5D-5L scores will first be converted into utilities using a pre-existing preference valuation set for EQ-5D health states of the Belgian (Flemish) adult population [59–61]. The resulting utility, a value between 0 (death) and 1 (full health), will then be transformed to DWs by subtracting it from the EQ-5D population averages for the same sex and age-group [52, 62, 63]. Since Lyme related mortality is exceptional and no Lyme related deaths have been reported in Belgium between 1998 and 2014 (only data available), YLLs will equal zero.

#### Costs

To minimize recall bias between two consecutive questionnaires (recall periods up to 12 months at the end of the follow-up period), patients are invited to keep a cost diary during the complete study period. The patients’ questionnaires assess direct and indirect non-medical costs (e.g. travel costs, absence from work, paid help) and provide information on direct medical costs related to medication use, consultations and hospitalizations (e.g. how many visits to a GP/specialist/…, use of over the counter medication). Additional details on some of these direct medical costs, which can’t be provided by the patients themselves, are collected through the follow-up questionnaires completed by the GPs (e.g. information on the laboratory tests). The standard unit costs (the price of medication, a consultation, a laboratory test, a hospitalization) will be obtained from official sources (e.g. Belgian centre for pharmacotherapeutic information (BCFI), National Institute for Health and Disability Insurance (RIZIV)). Supplementary data on hospitalization costs will be collected through the minimal hospital data (MZG), minimal financial data (MFG) and Belgian health insurance databases.

### Statistical analysis

All analyses will be performed in R [64]. A conditional log-binomial regression model with adjustment for comorbid illness will be used to compare the development of the non-specific disease symptoms between the different cohorts and the “matched” non-Lyme borreliosis control group in order to calculate the incidence of PTLDS in patients with Lyme borreliosis [65]. Within cohort 1 & 2, a multivariate log-binomial regression model will be used to calculate exposure risk ratio (RR) of the risk factors for development of PTLDS. For the case-control, the odds ratio (OR) determination for tick-borne co-infections as a risk factor will be done using a multivariate logistic regression. The multivariate models will include adjustment for potential confounding variables (age, gender and comorbid illness).

The burden of disease will be estimated in collaboration with the “Institut de recherche santé et société (IRSS) Université catholique de Louvain (UCL)” using the DALY package [56–58, 66]. The total costs (mean, median & confidence intervals) for each of the different clinical manifestations, including PTLDS, will be calculated by combining the different data sources. The cost analysis will be carried out in collaboration with the Centre for Health Economics Research & Modeling Infectious Diseases (CHERMID), University of Antwerp (UA).

## Discussion

Lyme borreliosis has been an increasingly “hot topic” in Belgium and the rest of the world, with uncertainties and intensive debates on the burden of the disease and on possible long-lasting (“chronic”) symptoms.

The incidence of PTLDS has been estimated in previous studies, but the reported results vary widely. This is likely related to differences in the inclusion criteria and PTLDS case definitions used, as well as to the variation among study designs [15]. The strength of the presented study lies in the combination of different methodologies which aim at collecting objective data with regard to PTLDS: the prospective study design allows collecting detailed information throughout the complete disease progression and minimizes recall bias; the inclusion of a non-Lyme borreliosis control group allows controlling for the background prevalence of aspecific symptoms in the general population; through the use of validated questionnaires the severity of the aspecific symptoms will be assessed (and afterwards compared) accurately. The availability of Belgian population norms for some of the validated questionnaires is an additional benefit. Furthermore, the use of parallel questionnaires for the patients and their GPs allows comparison between the different perspectives on the persistent aspecific symptoms as well as to obtain detailed information on possible risk factors (e.g. comorbidity, diagnosis, treatment, complications). By looking at both the prevalence and the severity of the symptoms occurring with PTLDS, as well as the impact on the patient’s life, we will be able to assess all components of the PTLDS case definition [13]. Different possible risk factors will be assessed through the patient questionnaires and the analyses of other tick-borne co-infections (in EM patient blood samples) allows collection of innovative data on the importance of co-infections on the clinical presentation and progression of Lyme borreliosis. A better comprehension of this syndrome and its risk factors will allow informing health care providers and patients about what to expect after treatment and, if possible, to early identify those patients at increased risk.

The estimation of the burden of disease, quantified by a composite health measure, can be used to compare and prioritize between the different clinical manifestations of Lyme borreliosis but also between different diseases and health interventions, in order to set public health priorities [67]. Some European projects assessed the burden of different communicable diseases but they did not include Lyme borreliosis [67, 68]. A study in the Netherlands did estimate the disease burden for Lyme borreliosis and found that the majority of the substantial burden is caused by persisting symptoms [69]. The additional benefit of our study for the burden estimation is again its prospective study design. In the end, the results will be used to inform the general public, patients associations, policymakers and health care providers on the actual disease burden related to the different stadia of Lyme borreliosis in Belgium.

A few European studies have examined the costs associated with Lyme borreliosis but often only part of the costs were included (e.g. costs of Lyme neuroborreliosis in Sweden [70], cost of testing [71] and of hospital care in Germany [72]). One study in Scotland included both direct and indirect costs for early and late Lyme borreliosis patients and follow-up costs [73]. These studies didn’t include the specific supplementary cost for patients with PTLDS although it is expected to contribute significantly to the overall costs of Lyme borreliosis. A recent American study showed that PTLDS-related diagnoses are associated with notably higher costs and health care utilization ($ 3798 higher costs and 66% more outpatient visits) as compared to Lyme borreliosis without PTLDS-related diagnoses [21, 74]. Through the inclusion of different costs (both direct and indirect, medical and non-medical) for both the patients and the health care system, this study will provide an overall view of the costs associated with Lyme borreliosis, its diagnosis, treatment, follow-up, etc.. The results could be used in future cost-effectiveness analyses of potential interventions.

A first possible limitation of the study is the work–load for the patients at the moment of inclusion (long questionnaire) which might keep them from participating. However, in the context of the current attention for Lyme borreliosis in the media and the debate on existing persisting symptoms, we believe we will find sufficient patients willing to participate to the study. Secondly, as prescribed in the Belgian guidelines for the diagnosis and treatment of Lyme borreliosis, diagnosis of an EM is solely based on clinical symptoms. This implies that the inclusion of EM patients in our study is dependent on correct EM recognition by the participating GPs. As a reminder, the clinical signs of an EM, possible differential diagnoses and corresponding pictures are provided to the participating GPs at the start of the study. The diagnosis of disseminated Lyme borreliosis is based on both clinical symptoms recognized by specialists and laboratory testing. Third, due to project time restrictions, we will only be able to follow up patients during a maximum of two years. It would have been interesting to follow up patients for a longer period. Many patients and patient associations indeed have questions about symptoms after a longer period after treatment. However a longer study period is likely to induce an important loss to follow-up over time. Finally, the study will not allow the collection of data with regard to a group of patients who attribute their persisting aspecific symptoms (fatigue, musculoskeletal pains and cognitive disorders) to Lyme borreliosis, but without having a confirmed diagnosis.

In conclusion, through its multidisciplinary approach, the HUMTICK prospective cohort study allows addressing multiple relevant research questions with regard to Lyme borreliosis; the enrollment of both EM patients (early localized Lyme) and disseminated Lyme borreliosis patients, together with the follow-up over time, will not only allow to estimate the incidence of PTLDS but also to estimate the disease burden and cost associated with the different manifestations of Lyme borreliosis, specifically in Belgium. In addition, the study design allows simultaneous evaluation of risk factors for the development of PTLDS and will so improve the understanding of the evolution of the different clinical manifestations of Lyme borreliosis after treatment.

## Additional file

Additional file 1: Specific description of the case definitions for confirmed cases of disseminated Lyme borreliosis which will be included in the HUMTICK study. (DOCX 19 kb)

## Abbreviations

BCFI: Belgian centre for pharmacotherapeutic information
CFQ: Cognitive failure questionnaire
CHERMID: Centre for Health Economics Research & Modeling Infectious Diseases
DALY: Disability-adjusted life year
DW: Disability weight
EM: Erythema migrans
GP: General practitioner
GALI: Global activity limitation index
IDSA: Infectious diseases society of America
MFG: Minimal financial data
MZG: Minimal hospital data
RIVM: National Institute for Public Health and Environment
NRC: National reference center
OR: Odds ratio
PCR: Polymerase chain reaction
PTLDS: Post-treatment Lyme disease syndrome
RIZIV: National Institute for Health and Disability Insurance
RR: Risk ratio
UA: University of Antwerp
UCL: Université catholique de Louvain
UZL: University Hospitals Leuven
WIV-ISP: Scientific Institute of Public Health
YLD: Years lived with disability
YLL: Years of life lost
