# Supplementary material for: The HUMTICK study: protocol for a prospective cohort study on post-treatment Lyme disease syndrome and the disease and cost burden of Lyme borreliosis in Belgium
Source: Arch Public Health. 2017 Aug 7;75:42. doi: 10.1186/s13690-017-0202-z (PMC5545865; doi:10.1186/s13690-017-0202-z)
Supplement: Additional file 1: — Specific description of the case definitions for confirmed cases of disseminated Lyme borreliosis which will be included in the HUMTICK study. (DOCX 19 kb) [file 13690_2017_202_MOESM1_ESM.docx]

**Additional file 1: Specific description of the case definitions for confirmed cases of disseminated Lyme borreliosis which will be included in the HUMTICK study:**

**Adapted from CDC and EUCALB case definitions* [1-2]**

- Isolation or positive PCR from tissue or body fluid

OR

- Positive serology (using the two-tier ELISA and Western Blot)

AND

at least one of the following clinical manifestations corresponding with disseminated Lyme borreliosis, when an alternate explanation is not found:

- **Skin manifestation:**

Multiple erythema migrans or acrodermatitis chronica atrophicans.

- **Musculoskeletal system (Lyme arthritis):**

Recurrent episodes (weeks or months) of objective joint swelling in one (commonly the knee) or a few joints, sometimes followed by chronic arthritis in one or a few joints. The following manifestations are not considered for inclusion: chronic progressive arthritis not preceded by brief attacks, chronic symmetrical polyarthritis, and arthralgia, myalgia, or fibromyalgia syndromes alone.

- **Nervous system (neuroborreliosis):**

Any of the following neurological symptoms (alone or in combination): lymphocytic meningitis; cranial neuritis, particularly facial palsy (uni- or bilateral); radiculitic pain; or, rarely encephalomyelitis, confirmed by demonstration of antibody production against *B. burgdorferi* in the cerebrospinal fluid (CSF), evidenced by a higher titer of antibody in CSF than in serum (positive antibody index). Headache, fatigue, paresthesia, or mildly stiff neck alone, are not considered for inclusion.

- **Cardiovascular system (carditis):**

Acute onset of high-grade (2nd-degree or 3rd-degree) atrioventricular conduction defects that resolve in days to weeks and are sometimes associated with myocarditis. Palpitations, bradycardia, bundle branch block, or myocarditis alone are not considered for inclusion.

* Belgium is an area endemic for Lyme Borreliosis, therefore all included patients fulfill the criteria of exposure to *B. burgdorferi.*

Reference List

1. Stanek G, Fingerle V, Hunfeld KP, Jaulhac B, Kaiser R, Krause A *et al*.: Lyme borreliosis: clinical case definitions for diagnosis and management in Europe. *Clin Microbiol Infect* 2011, **17:** 69-79.

2. Lyme Disease *(Borrelia burgdorferi)* 2011 Case Definition. https://wwwn.cdc.gov/nndss/conditions/lyme-disease/case-definition/2011/. Accessed 11 Oct 2016.
